# Care Groups I: An Innovative Community-Based Strategy for Improving Maternal, Neonatal, and Child Health in Resource-Constrained Settings

**DOI:** 10.9745/GHSP-D-15-00051

**Published:** 2015-09-10

**Authors:** Henry Perry, Melanie Morrow, Sarah Borger, Jennifer Weiss, Mary DeCoster, Thomas Davis, Pieter Ernst

**Affiliations:** ^a^​ Johns Hopkins Bloomberg School of Public Health, Baltimore, MD, USA; ^b^​ ICF International (Maternal and Child Survival Program), Washington, DC, USA; ^c^​ Food for the Hungry, Washington, DC, USA; ^d^​ Concern Worldwide/US, New York, NY, USA; ^e^​ Feed the Children, Oklahoma City, OK, USA; ^f^​ World Relief/Mozambique, Chokwe, Mozambique

## Abstract

In view of the slow progress being made in reducing maternal and child mortality in many priority countries, new approaches are urgently needed that can be applied in settings with weak health systems and a scarcity of human resources for health. The Care Group approach uses facilitators, who are a lower-level cadre of paid workers, to work with groups of 12 or so volunteers (the Care Group), and each volunteer is responsible for 10–15 households. The volunteers share messages with the mothers of the households to promote important health behaviors and to use key health services. The Care Groups create a multiplying effect, reaching all households in a community at low cost. This article describes the Care Group approach in more detail, its history, and current NGO experience with implementing the approach across more than 28 countries. A companion article also published in this journal summarizes the evidence on the effectiveness of the Care Group approach. An estimated 1.3 million households—almost entirely in rural areas—have been reached using Care Groups, and at least 106,000 volunteers have been trained. The NGOs with experience implementing Care Groups have achieved high population coverage of key health interventions proven to reduce maternal and child deaths. Some of the essential criteria in applying the Care Group approach include: peer-to-peer health promotion (between mothers), selection of volunteers by mothers, limited workload for the volunteers, limited number of volunteers per Care Group, frequent contact between the volunteers and mothers, use of visual teaching tools and participatory behavior change methods, and regular supervision of volunteers. Incorporating Care Groups into ministries of health would help sustain the approach, which would require creating posts for facilitators as well as supervisors. Although not widely known about outside the NGO child survival and food security networks, the Care Group approach deserves broader recognition as a promising alternative to current strategies for delivering key health interventions to remote and underserved communities.

## Abstract (precis)

Care Groups use volunteers to motivate mothers to adopt key MCH behaviors. The volunteers meet as a group every 2–4 weeks with a paid facilitator to learn new health promotion messages. Key ingredients of the approach include: peer-to-peer health promotion, selection of volunteers by the mothers, a manageable workload for the volunteers (no more than 15 households per volunteer), frequent (at least monthly) contact between volunteers and mothers, and regular supervision of the volunteers.

## INTRODUCTION

There is a recognized need to accelerate progress in reducing maternal and child mortality in the 75 countries of the world where 95% of the world’s maternal and child deaths take place.^1^^-^^3^ The Millennium Development Goals (MDGs), established in the year 2000, called for achieving by the year 2015 reductions of three-fourths and two-thirds, respectively, in maternal and child mortality based on 1990 levels.^4^ These goals will not be achieved by the great majority of these countries, particularly in sub-Saharan Africa, where only 5 of 44 countries are on track to achieve the maternal health MDG and only 14 are on track to achieve the child health MDG.^5^

One of the important reasons for lack of progress has been the low population coverage of key interventions proven to be effective for reducing maternal and child deaths. Although the median population coverage of immunizations and vitamin A supplementation is in the range of 80%, coverage of other key interventions is 60% or less, and for a number of interventions, the median range of coverage is 30% or less.^5^ In some countries, levels of coverage are less than 10%.^5^

There is a lack of evidence that facility-based services by themselves in resource-constrained settings with high mortality can achieve high levels of population coverage of key maternal and child health interventions and mortality impact, and some evidence that they cannot.^6^^-^^8^ Expanding coverage of key interventions and achieving documented reductions in maternal, neonatal, and child mortality will require approaches that are not only low-cost and effective on a short-term, pilot basis in small populations but also low-cost, effective, and feasible at scale over the longer term. This requires, among other things, approaches that engage the community as partners, empower women and communities, and reach a high proportion of households with health education that encourages healthy behaviors and appropriate use of health facilities.^9^

Engaging communities will be necessary to expand coverage of key maternal and child health interventions around the world.

Interest in and experience with community health workers (CHWs) is growing rapidly, and CHW programs are expanding in many countries.^10^^,^^11^ CHW programs in some countries (such as Bangladesh, Brazil, Ethiopia, and Nepal) have been widely credited with achieving high levels of population coverage of key maternal and child health interventions and marked improvements in child survival, while in other countries such as India and Pakistan progress has lagged behind in spite of large-scale CHW programs.^5^ There is a wide diversity of CHW programs and cadres, with some using “professionalized” workers with 1 year or more of training, a broad scope of preventive and curative skills, and a full-time government salary while others engage volunteers working only a few hours a week on a highly specialized activity such as immunizations, HIV/AIDS control, or distribution of bed nets. In all cases, CHWs are formally trained and engaged with the health system but work at the community level outside of facilities and receive no formal professional or paraprofessional certificate and no tertiary education degree.

This article describes the Care Group approach—a delivery strategy for expanding coverage of maternal and child health interventions using volunteer CHWs. We describe what Care Groups are, the key ingredients (the “secret sauce”) that seem to be important for their successful implementation, their history, the field experience with use of this delivery strategy, modifications that are emerging in Care Group implementation, and how Care Groups might be integrated into government health programs. Finally, we highlight Care Groups as one example of the growing importance of participatory women’s groups in improving maternal and child health. A companion article in *Global Health: Science and Practice* summarizes the evidence on the effectiveness, cost, and cost-effectiveness of Care Groups to improving child survival.^12^

Care Groups are a community-based strategy for expanding coverage of MCH interventions using volunteer health workers.

## WHAT ARE CARE GROUPS?

The formal definition of a Care Group is the following^13^:

A Care Group is a group of 10–15 volunteer, community-based health educators who regularly meet together with project staff for training and supervision. They are different from typical mother’s groups in that each volunteer is responsible for regularly visiting 10–15 of her neighbors, sharing what she has learned and facilitating behavior change at the household level. Care Groups create a multiplying effect to equitably reach every beneficiary household with interpersonal behavior change communication.

A representation of a Care Group intervention delivery system is shown in the Figure. The system is established initially by identifying 1 volunteer (called a Care Group Volunteer) who is responsible for about 12 mothers (pregnant women and mothers of young children, usually 0–59 months of age or 0–23 months of age). The Care Group Volunteer is often selected by the mothers themselves; sometimes community leaders participate in the selection process. Supervisory field staff are recruited and trained to set up Care Groups in collaboration with community leaders so that: (1) Care Group Volunteers are in place and are responsible for about 12 mothers who are their neighbors, and (2) all pregnant women and mothers of young children are linked to a Care Group Volunteer.

Care Group Volunteers are often selected by the mothers themselves.

**FIGURE:**
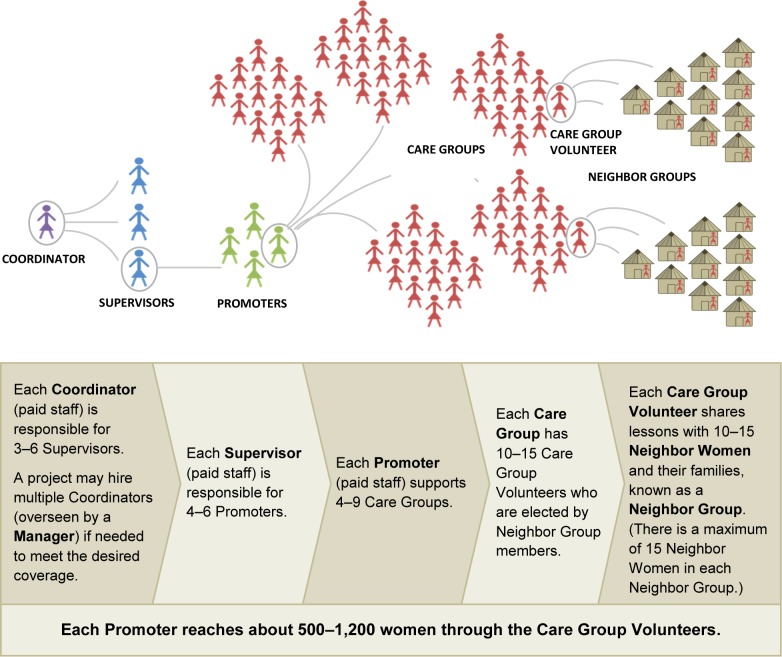
Structure of a Typical Care Group Delivery Strategy

Depending on the size of the population covered by the project or program, several layers of paid program staff are required so that a Care Group Facilitator (referred to in the Figure as a Promoter and who is a low-level paid project staff person) can meet with each Care Group every 2–4 weeks for 2 hours or so. At that time, the Care Group Facilitator teaches one or a small number of health promotion messages for the Care Group Volunteers to share with the women for whom they are responsible. The Facilitator uses participatory learning, including role play and composition of songs and skits, to convey messages. During the following 2–4 weeks (depending on the schedule established by the program), each Care Group Volunteer meets with each of the women for whom she is responsible (and other family members who may be present, such as grandmothers, husbands, and older children)—either by visiting the woman at her home or by meeting with her and a few neighbors as a small group. At the subsequent Care Group meeting, the Care Group Volunteers discuss their experience in sharing the previous messages and learn a new set of messages. In most Care Group programs, the Care Groups Volunteers also report births and deaths to the Care Group Facilitators/Promoters, who report this information upward through the health information system. (A manual outlining how to monitor mortality using the Care Group approach is available at: http://www.coregroup.org/resources/271-the-mortality-assessment-for-health-programs-system.)

The Facilitators/Promoters along with higher-level supervisory staff meet together every few months to learn the health promotion messages that they will later convey to the Care Group Volunteers. The Facilitators/Promoters are also taught participatory methods for behavior change promotion, including demonstrations, role plays, stories, and songs (often composed by the Care Group Volunteers themselves) to convey these messages.

The educational content focuses on key knowledge about maternal and child health, important household practices for promoting maternal and child health, and indications for use of health facilities, including danger signs for which medical care should be sought. Messages are often based on results of formative research such as positive deviance studies and barrier analysis^14^ studies that identify behavioral determinants of key behaviors.

Further details regarding what are considered to be essential criteria for the Care Group approach are shown in Box 1. Additional suggested criteria are shown in Box 2. The rationale for these lists is described in greater detail elsewhere.^13^ The degree to which each criterion in Box 1 and Box 2 is required for the Care Group approach to be effective and whether or not the approach would function if one or more criteria were not met is unknown at present, but fidelity to most, if not all, criteria seems to be essential at this stage in the development of the approach. Over time, with further experience and evaluation, variations in the approach could be tested. Box 3 provides a case study of how one Care Group project in Mozambique functioned.

**BOX 1. Essential Criteria for Application of the Care Group Approach**

1. Peer-to-peer (mother-to-mother for maternal and child health and nutrition behaviors) health promotion is essential.
2. Care Group Volunteers are selected by mothers within the group of households they will serve or by the leadership in the village.
3. Because they are volunteers, the Care Group Volunteers must limit their workload. There should be no more than 15 households per Care Group Volunteer. The terrain and distance between households may affect the number of households for which Care Group Volunteers can take responsibility. In settings where households are dispersed or the terrain is difficult to traverse, Care Group Volunteers may need to take responsibility for fewer households.
4. There should be between 6–16 members in a Care Group.
5. Contact between the Care Group Volunteer and her assigned beneficiary mothers is monitored; contact should occur at a minimum once a month, but preferably twice monthly.
6. The plan is to reach 100% of the households in the targeted group on at least a monthly basis and to attain at least 80% monthly coverage of households within the target group. Coverage is monitored.
7. Care Group Volunteers collect vital events (data on pregnancies, births, and deaths). This is very important as they can discuss and solve problems as a group related to what kind of follow-up is needed and how similar deaths might be prevented in the future.
8. The majority of what is promoted through the Care Group is directed toward reduction of maternal and child mortality and malnutrition (e.g., Essential Nutrition Actions and Essential Hygiene Actions). It could be a useful strategy to include other topics but this is what Care Groups were originally intended to do.
9. Care Group Volunteers should use some sort of visual teaching tool (e.g., job aids, flip charts) for health promotion at the household level.
10. Participatory methods of behavior change communication are important. This is not specific only to Care Groups, but Care Groups should use best practices for behavior change.
11. No more than 1–2 hours should be spent in a meeting of Care Group Volunteers. Such “drip training” involves small amounts of information, and then the information is applied. The Care Groups can fit the time needed for the meeting into their schedule. Care Group Volunteers then bring the information and messages to the women in their catchment area as they have time.
12. Supervision of Promoters and of at least one of the Care Group Volunteers should occur at least monthly. This is an important part of the Care Group approach and part of the cascade.
13. All of a Care Group Volunteer’s beneficiaries should live less than a 1-hour’s walk from the Volunteer’s home.
14. The implementing agency needs to successfully create a project/program culture that conveys respect for women, for the Care Group Volunteers, and for the beneficiaries.

**BOX 2. Suggested Additional Criteria for Optimal Functioning of the Care Group Approach**

1. Formative research should be conducted, especially on the key behaviors that will be promoted. If the key behaviors promoted are not the leading causes of child death in the program area, an impact on mortality may not be achieved.
2. Care Group Promoters are paid staff who meet with and directly train Care Group Volunteers. Care Group Promoters should have no more than 9 Care Groups for which they are responsible. This is particularly important when Care Groups meet every 2 weeks. For a Care Group Promoter to develop a personal relationship and really know those with whom s/he is working, the Promoter should not work with more than about 150 people. This can be achieved if a Care Group Promoter is not responsible for more than 9 Care Groups. A Care Group should have at the most 15 members.
3. Measurement of many of the results-level indicators should be conducted annually at a minimum. This is achieved by carrying out a survey of a random sample of households in the project or program area. Assuming households are selected at random, 96 households are needed for the survey. Supervisory staff can carry out this survey in the course of their regular field supervision activities. These data are needed to manage any program well.
4. Differences between Care Group Promoters and Care Group Volunteers in their social characteristics and in educational levels should not be too great. For example, an extreme difference is when a Care Group Promoter has a bachelor-level university degree and her Care Group Volunteers are illiterate. A great social distance makes it difficult for Care Group Promoters to connect with Care Group Volunteers in an effective way.
5. The “care” in Care Groups may not be quantifiable, but it is supremely important for an entire system based on volunteerism. Care and respect need to be modeled from the senior leadership all the way through to the volunteers.
6. Ensuring that each Care Group Volunteer does not have more than 3–4 hours of work per week is essential in order not to overload and overburden her.
7. Peer selection of Care Group Volunteers helps to identify the most effective recruits.
8. Field Supervisors and Care Group Facilitators/Promoters need support from their supervisors on a regular and intensive basis, and they need to be provided with the tools to make their work effective, including transport and educational materials/visual tools such as flip charts.
9. Participatory methods of behavior change communication should be used, including songs, role play, games, and stories. Oftentimes Care Groups make up their own songs to use.
10. Flexible management and supervision that enable the Care Group principles to be upheld while adjusting to local contextual realities and implementation hurdles is recommended. The number of Field Supervisors and Care Group Promoters as well as the number of Care Group members needs to be adjusted based on local geographical conditions and ease of transport.

**BOX 3. An Illustrative Care Group Child Survival Project in Mozambique Implemented by Food for the Hungry**

In 5 districts of Mozambique with a total population of 1.1 million people, the Care Group project recruited 5 Field/District Supervisors (with three Supervisors serving one district each, and two Supervisors serving two districts each), each with technical or professional training, such as in nursing or as a medical technician. Each District Supervisor recruited about 13 Promoters/Facilitators (for a total of 65 Promoters) by working with community leaders to identify candidates living in the area with at least 5 years of education and who could read and write and perform simple mathematical calculations.

With the help of community leaders and teachers, the District Supervisor working with each Promoter registered all the pregnant women and women with a child 0–23 months of age as “beneficiary mothers.” These women selected Leader Mothers (Care Group Volunteers). A total of 325 Care Groups with 4,095 Leader Mothers were created: 5 Care Groups for each Promoter and 12–13 Leader Mothers per Care Group.

All the District Supervisors and Promoters met together to learn the first module to be taught to the Care Groups, entitled “Working Together with Communities.” Each module had 4–5 lessons. The District Supervisors and Promoters met together 3–4 times a year to learn new modules. A sample of training materials and training aids can be found in Annexes 13 and 14 of the project’s final evaluation, located at: http://caregroups.info/docs/FH_Final_Eval_Report_27Dec2010.pdf.

Every 2 weeks, each Promoter met with each of his/her 5 Care Groups. Each Care Group meeting lasted about 1.5 to 2 hours. At each meeting, 15–20 minutes were spent reviewing the previous activities since the last Care Group meeting 2 weeks previously. The Leader Mothers also informed the Promoter of any births or deaths that had occurred during the previous 2 weeks. Then they learned 3-4 new key messages to share with their households and practiced among themselves how to best present them to the beneficiary mothers.

Over the next 2 weeks, the Leader Mothers met with each of their 12 or so beneficiary mothers, either individually or in small groups. Over time, these beneficiary mothers “graduated” as their child reached 2 years of age, and newly pregnant women or those with a newborn became a beneficiary mother. Leader Mothers used a flip chart to illustrate the lesson being given.

When it became apparent that neonatal deaths were frequent, the Leader Mothers began to visit newborns as soon as possible after birth and then on a daily basis during the first week, 3 times a week during the second week, twice during the third week, and once during the fourth week. Leader Mothers received training in danger signs of neonates, used a checklist, and counseled mothers during these visits using flip charts and a checklist for newborns.

One Leader Mother in each Care Group was selected as a C-IMCI Leader Mother and received 5 days of training. C-IMCI refers to Community-based Integrated Management of Childhood Illness. She served as a resource and a referral point for the other Leader Mothers in the Care Group.

The project had only one vehicle, which was used to transport the project leadership team from the project headquarters in the city of Beira to and from the project area. Each of the 5 District Supervisors had a motorbike and each of the Promoters had a bicycle.

## HOW DID CARE GROUPS EMERGE?

The Care Group approach was first developed in 1995 in the Guija and Mabalane districts of Gaza Province in Mozambique by staff members of World Relief (Pieter Ernst and Muriel Elmer, later with support from Warren and Gretchen Berggren) as they were developing an implementation plan for a child survival project funded by the United States Agency for International Development (USAID) Child Survival and Health Grants Program. This program proved to be highly successful in achieving impressive gains in coverage of key child survival interventions. Two years later, in 1997, after receiving training from World Relief, Food for the Hungry initiated a Care Group project in the Sofala Province of Mozambique under the leadership of Tom Davis and with funding from the USAID Title II Food for Peace program. This was the first replication of the model by another organization; the project achieved substantial decreases in moderate and severe stunting. A second World Relief Mozambique child survival project, implemented between 1999 and 2003 in the Chokwe district of Gaza Province (excluding the town of Chokwe), was similarly successful; its impact on under-5 mortality, as estimated by expansions of coverage of key maternal and child health interventions using the Lives Saved Tool (LiST), was one of the highest achieved up to that time by USAID-supported child survival projects.^15^ Later, the CORE Group provided a grant to carry out an independent assessment of the mortality impact of the project and to prepare a manual describing the Care Group delivery strategy.^16^

As evidence of the effectiveness and feasibility of Care Groups began to accumulate among NGO child survival projects, other NGOs began to try the approach. Again, under the leadership of Tom Davis, Curamericas Global in Huehuetenango, Guatemala implemented the Care Group approach. World Relief, Food for the Hungry, and Curamericas Global also began to apply the Care Group approach at their other project sites in different countries. The success of these projects, the technical support offered to other organization by Melanie Morrow (then working with World Relief) and Tom Davis (then working with Food for the Hungry), and the availability of a very useful implementation guide^16^ all fueled adoption of the approach by other NGOs funded by the USAID Child Survival and Health Grants Program. (A recently updated version of the implementation guide is available at: http://www.fsnnetwork.org/care-groups-training-manual-program-design-and-implementation.) Other early adopters of the Care Group approach included the American Red Cross in Cambodia, Plan International in Kenya, the Salvation Army World Service Office in Zambia, Concern Worldwide in Burundi, Medical Teams International in Liberia, and Catholic Relief Services in Malawi.

The fact that all the early Care Group projects were funded by the USAID Child Survival and Health Grants Program meant they all had baseline measurements of population coverage of key interventions obtained from household surveys as well as end-of-project measures using similar survey instruments since this was required of all grantee projects. The projects were therefore able to assess changes in practice and coverage over the course of the projects. This success was shared with the NGO community through a workshop led by World Relief in Mozambique in 2005 and through presentations at CORE Group meetings, leading to adoption of the Care Group strategy by a number of other NGOs with funding from a variety of sources in addition to the USAID Child Survival and Health Grants Program.

Because all early Care Group projects were funded by USAID, they all had both baseline and endline outcome measurements obtained from household surveys.

## WHAT IS THE EXPERIENCE SO FAR WITH CARE GROUP IMPLEMENTATION?

A small number of NGOs working in a variety of countries and settings continued to successfully achieve high levels of intervention coverage in populations of 100,000–200,000 people. (See the companion paper in *Global Health: Science and Practice* for details about effectiveness of the Care Group approach.^12^) And with this, enthusiasm for the Care Group approach began to grow. In 2010, 14 NGOs had implemented Care Group projects in 16 countries. Only 5 years later, that number had grown to 25 NGOs in 28 different countries in all regions of the world (Box 4). In all cases, NGOs have implemented Care Groups in collaboration with ministry of health (MOH) programs and in accordance with MOH policies and strategies. And in all cases, Care Group projects promote use of MOH services and programs, including facility-based services. At present, based on available information, we estimate that 1.3 million households have been reached using the Care Group implementation system, and at least 106,000 Care Group Volunteers have been trained.

Care Group projects in 28 countries have reached an estimated 1.3 million households, mostly in rural areas.

**BOX 4. Organizations and Countries With Experience Implementing Care Groups as of 2015**

**Organizations with experience implementing Care Groups:**

1. ACDI/VOCA (http://acdivoca.org/)
2. ADRA (https://adra.org/)
3. Africare (https://www.africare.org/)
4. American Red Cross (http://www.redcross.org/)
5. CARE (http://www.care.org/)
6. Concern Worldwide (https://www.concern.net/)
7. Catholic Relief Services (http://www.crs.org/)
8. Curamericas (http://www.curamericas.org/home)
9. Emmanuel International (www.eim-us.org/)
10. Feed the Children (http://www.feedthechildren.org/)
11. Food for the Hungry (http://fh.org/)
12. Future Generations (http://www.future.org/)
13. GOAL (https://www.goalglobal.org/)
14. International Aid (http://www.internationalaid.org/)
15. International Medical Corps (https://internationalmedicalcorps.org/)
16. Living Water International (http://www.water.cc/)
17. Medair (http://relief.medair.org/en/)
18. Medical Teams International (http://www.medicalteams.org/)
19. PLAN (http://plan-international.org/)
20. Project Concern International (http://www.pciglobal.org/)
21. Salvation Army World Service Office (http://www.sawso.org/)
22. Save the Children (http://www.savethechildren.org/)
23. World Renew (http://www.worldrenew.net/)
24. World Relief (http://worldrelief.org/)
25. World Vision (http://www.worldvision.org/)

**Countries in which Care Group projects have been or are currently being implemented:**

1. Bangladesh
2. Bolivia
3. Burkina Faso
4. Burundi
5. Cambodia
6. Democratic Republic of the Congo
7. Ethiopia
8. Guatemala
9. Haiti
10. Indonesia
11. Kenya
12. Liberia
13. Malawi
14. Mexico
15. Mozambique
16. Nicaragua
17. Niger
18. Peru
19. Philippines
20. Rwanda
21. Senegal
22. Sierra Leone
23. Somalia
24. South Sudan
25. Sudan
26. Uganda
27. Zambia
28. Zimbabwe

Source: Care Groups Info.^17^

Almost all the Care Group projects implemented so far have been in rural areas of low-income countries. To our knowledge, there is only one example of Care Group implementation in an urban or peri-urban setting,^18^ although several rural projects have had “pockets” of peri-urban populations. Projects often vary in terms of the specific interventions implemented (such as nutrition, diarrhea control, newborn health, immunizations, and so forth) depending on the local epidemiological context. The details of supervision and training also vary from project to project and from NGO to NGO.

International NGOs have initiated implementation of all known Care Group projects to date. The major donors for these projects have been the USAID Child Survival and Health Grants Program, in-country USAID missions, the USAID Food for Peace (Title II) programs, and the USAID Office of U.S. Foreign Disaster Assistance. However, donor support has expanded to include the World Bank (for Care Group projects in Malawi and Mozambique), the Canadian International Development Agency (CIDA), the British Department for International Development (DfID), the European Commission’s Humanitarian Aid and Civil Protection department (formerly called the European Community Humanitarian Aid Office and still referred to as ECHO), the United Nations Children’s Fund (UNICEF), and private NGO funds.

There is now early experience in applying the Care Group delivery strategy within MOH rural health care delivery systems. Concern Worldwide has carried out an operations research project in Burundi comparing the effectiveness of the traditional NGO Care Group project structure (in which the Care Group Facilitators/Promoters are paid by the NGO) with an alternative approach in which Care Group Facilitators/Promoters are MOH CHWs (who are unpaid volunteers). The findings indicate that—with NGO technical and financial support during the time of the study—the effectiveness of the Care Group strategy using MOH CHWs as Care Group Facilitators/Promoters is similar to the traditional NGO implementation of the approach.^19^ In this case, the MOH CHWs served as Care Group Facilitators/Promoters in addition to their usual duties, but they each supervised only 2 Care Groups rather than the usual 5–9 Care Groups in the typical NGO implementation. (A short user’s guide on how to integrate Care Groups into MOH health systems is available at http://www.fsnnetwork.org/sites/default/files/resource_uploads/integrating_care_groups_into_moh_systems_a_users_guide.pdf.)

A project in Burundi has tested the feasibility of embedding Care Group Facilitators within the Ministry of Health.

**Figure:**
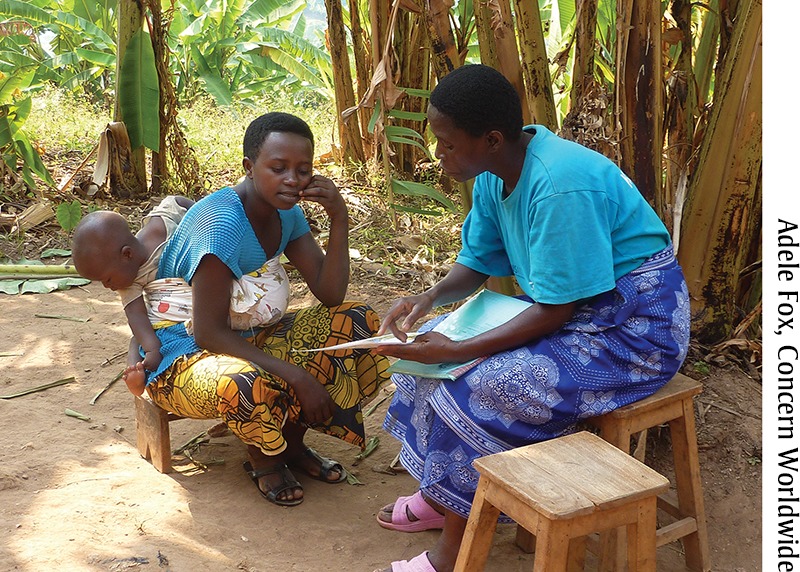
A Care Group Volunteer in Burundi shares a health message during a home visit. Care Group Volunteers make home visits to each woman for which they are responsible on a monthly or twice-monthly basis.

## OTHER EMERGING MODIFICATIONS OF THE CARE GROUP APPROACH

One important example of a modified Care Group approach is in Rwanda.^20^ There, 3 international NGOs (Concern Worldwide, The International Rescue Committee, and World Relief) worked with the MOH to modify the Care Group delivery system to fit within the current role and functions of government CHWs (where the CHWs are volunteers who receive performance-based incentives). In this case, there were 4 CHWs in each village:

- Two (one male and one female) were assigned to carry out integrated community case management of childhood illness
- Another was responsible for maternal health (a female CHW)
- The fourth was responsible for social affairs (either a male for female)

The 4 CHWs were responsible together for the entire village of 60–80 households, but each focused on his/her specialty area. The Care Group methodology was modified so that the CHWs from 2–5 neighboring villages were organized into CHW Peer Support Groups with each group having up to 20 CHW members, about half of whom were men. The CHWs divided up households so each CHW was responsible for 15–20 households, and they visited each household monthly to provide behavior change communication. The project helped to organize and oversee training of the CHW Peer Support Groups in behavior change interventions. CHW Peer Support Groups elected unpaid CHW Cell Coordinators to help with the training and supervision of the CHW Peer Support Groups.

There were about 100 CHWs working in a health center’s catchment area. Each health center had an MOH employee in charge of supervising the CHWs working in the health center’s catchment area. This Community Health In-Charge at the health center also functioned as a CHW Peer Support Group Facilitator. This approach was implemented in a catchment area of 1.7 million people, reaching 18% of Rwanda’s population. Appropriate treatment for malaria, point-of-use water purification, and the proportion of caretakers who increased fluid or food during diarrheal episodes doubled or almost doubled.^21^ Care seeking for respiratory symptoms more than tripled, and skilled attendance during childbirth increased from 39% to 91%.^21^ Care seeking for fever, diarrhea, and symptoms of pneumonia in children were significantly greater in the catchment area with CHW Peer Support Groups than in the non-project districts.^20^

Another example of a modified Care Group approach is in Mozambique, where World Relief has just completed a 5-year Care Group project focused on tuberculosis (TB) control in the same locale where Care Group projects had been previously implemented. For the TB project, World Relief used many of the same Care Group Volunteers and similar supervisory structures as those that had been established over the previous 2 decades in 6 districts of Gaza Province. The project was able to achieve marked improvements in awareness about TB, its treatable nature, and the availability of free treatment at government health centers.^22^

## INTEGRATION OF CARE GROUPS INTO MINISTRY OF HEALTH DELIVERY SYSTEMS

As previously mentioned, the field experience with Care Groups has been primarily with projects implemented by NGOs. Opportunities for incorporating the Care Group approach into existing MOH programs would help give the approach a long-term, sustainable “home” that NGOs are not usually able to provide. There is room for new creative partnerships between NGOs and MOHs to implement the Care Group approach, from contracting out service delivery to engaging NGOs for training, monitoring, or quality assurance.

Incorporating the Care Group approach into Ministries of Health would help make it sustainable.

The essential link needed in MOH programs is the creation of formal postings for what we refer to in the Figure as Promoters (who meet with, teach, and support Care Group Volunteers) and Supervisors (who meet with, teach, and support the Facilitators/Promoters). Simply adding the duty of the Care Group Facilitator/Promoter to the existing duties of currently functioning CHWs would seem to be fraught with high risk of failure, as would adding the duty of the Supervisor to the MOH supervisor of MOH CHWs since these persons are already likely overburdened with too many responsibilities. However, the Concern Worldwide experience in Burundi revealed that in a situation in which CHWs were already overloaded with responsibilities that included community mobilization, integrated community case management (for pneumonia, diarrhea, and malaria), and home visits, giving them responsibility for supervising Care Groups actually lightened their workload—better prevention led to fewer cases and earlier home-based treatment and, at the same time, earlier referral led to fewer seriously ill children for CHWs to manage.^19^

**Figure:**
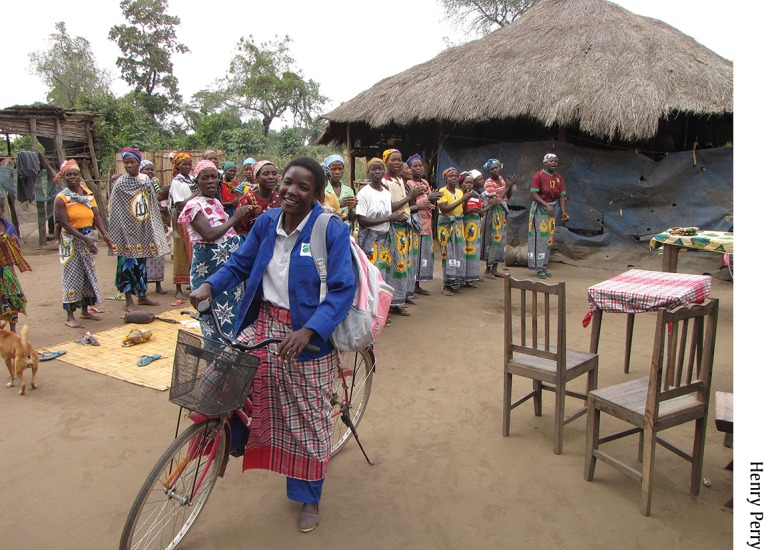
A Care Group Promoter from Mozambique meets with the volunteers she oversees to teach them new health promotion messages to share with their beneficiary mothers. The project provided each Care Group Promoter with a bicycle to facilitate supervision visits.

One option—aside from creating new posts specifically for these functions (which we acknowledge is quite difficult within the government system)—might include recruiting more MOH CHWs and supervisors of CHWs so that the additional Care Group workload would be manageable. Whatever strategy might be adopted, there would need to be additional resources devoted to high-quality, community-based delivery. Even though the costs of Care Group programs are quite modest (as is discussed in the companion article^12^), the success of Care Group implementation rests in large part on having well-trained, highly motivated, and well-supported field workers.

## CARE GROUPS AS AN EXAMPLE OF PARTICIPATORY WOMEN’S GROUPS

Care Groups are an example of how programs are gradually learning to harness the power of women working together to improve their own health and the health of their children. Women’s groups have been in use now for decades, but well-delineated methods for engaging them and mobilizing them to deliver key evidence-based interventions that result in scientifically demonstrated improvements in either population coverage of these interventions or improved population-level health outcomes have been lacking until recently.

A similar but nonetheless distinct approach to engaging the power of groups of women is women’s participatory learning and action (PLA) groups. In this approach, a facilitator meets with women in a village, and together they discuss health recommendations for pregnancy, birth, and neonatal care and how they could apply them in their own particular situation.^23^ Although pregnant women and those with newborns are targeted, anyone in the village can attend the meetings. This approach has benefited from rigorous implementation research in a variety of settings, all being led initially by the same research group based at the University of London. Robust evidence finds the approach can reduce maternal and neonatal mortality if there are an adequate number of facilitators to ensure high levels of service coverage.^24^ A key difference between Care Groups and PLA Groups is that with Care Groups, there is a systematic approach to reaching every household where there is a pregnant woman or young children with specific, carefully crafted messages. With PLA Groups, the focus is on the PLA Group discussing key health messages, formulating how they might incorporate these messages in their context, and then, through spontaneous dissemination, engaging other women who do not attend the facilitated sessions.^25^

Most certainly, other approaches are emerging now or will emerge in the future to harness the potential of participatory women’s groups. The enthusiasm for and the demonstrated results of Care Groups and PLA Groups indicate this is a fruitful area for further field experimentation with rigorous evaluation and broader implementation.

## CONCLUSIONS

Although not widely known about outside of NGO child survival and food security networks, Care Groups are a rapidly growing innovative approach to implementing maternal, neonatal, and child health and nutrition interventions. Care Groups are able to motivate women volunteers to assist their neighbors in adopting positive health behaviors and seeking health care from the formal health system when needed. The NGOs that have implemented the Care Group approach in a variety of field settings throughout the world have been uniformly enthusiastic about the effectiveness of the approach in changing behaviors, improving appropriate health care utilization, achieving demonstrable benefits in the health of mothers, neonates, and children, and empowering women and their communities. The Sustainable Development Goals, scheduled for adoption at the 2015 meeting of the United Nations General Assembly, call for the achievement of universal access to quality essential health services and the end of preventable deaths of newborns and under-5 children by the year 2030.^26^ Care Groups and related approaches hold great promise in helping to achieve these goals.

## Notes

**Peer Reviewed**
